# Angiofibromatoid histiosarcoma of the pulmonary artery

**DOI:** 10.1093/jscr/rjab132

**Published:** 2021-04-24

**Authors:** James Farag, Nicholas McNamara, Tristan D Yan

**Affiliations:** 1 Cardiothoracic Surgical Department, Royal Prince Alfred Hospital, Sydney, Australia; 2 Baird Institute of Applied Heart and Lung Research, Sydney, Australia; 3 Faculty of Health and Medicine, University of Sydney, Sydney, Australia

## Abstract

We describe the surgical management of a rare pulmonary angiomatoid fibrous histiosarcoma (AFH). A 62-year-old lady presented with shortness of breath and found to have a large left main pulmonary artery defect that was positron emission tomography-avid.

Following discussion in a thoracic multidisciplinary team meeting it was deemed unsafe to biopsy considering its intravascular position. The patient proceeded to theatre for a left pneumonectomy. She was successfully discharged home by Day 7. On follow-up the patient is well, and free of malignancy.

AFH is an incredibly rare form of sarcoma, and in particular in the thoracic region. We have demonstrated successful oncological resection of a rare intravascular lesion in the pulmonary artery.

## INTRODUCTION

We describe the surgical management of a rare pulmonary angiomatoid fibrous histiosarcoma (AFH). A 62-year-old lady presented with shortness of breath and found to have a large left main pulmonary artery defect that was positron emission tomography (PET)-avid. AFH is an incredibly rare form of soft tissue tumour of intermediate grade, and accounts for 0.3% of all soft tissue tumours [1]. It was first described by Enzinger *et al.* in 1979, and has since been defined as distinct to malignant fibrous histiocytoma [2]. It typically affects children and young adults, and predominantly arises in the deep dermis or subcutaneous tissue of the extremities, rarely ever occurring outside of the somatic soft tissues [3]. It is particularly infrequent within the thoracic cavity, and has rarely been described in the pulmonary areas [4, 5]. There has only been one report thus far describing AFH arising within a large vessel, and was of pulmonary artery origin also [6]. This is the second such report of a pulmonary vascular origin for AFH, and one of eight previously reported pulmonary AFH cases world-wide [1, 4, 5, 7, 8].

## CASE REPORT

Following discussion in a thoracic multidisciplinary team meeting it was deemed unsafe to biopsy considering its intravascular position. The patient proceeded to theatre for a left pneumonectomy. She was successfully discharged home by Day 7. On follow-up the patient is well, and free of malignancy. A 62-year-old lady with a past history of sarcoma presented with shortness of breath at rest and minimal exertion. A computed tomography pulmonary angiogram (CTPA) revealed an ovoid filling defect in her left main pulmonary artery, which did not resolve after 3 months of therapeutic anticoagulation on follow-up CTPA. A PET was performed demonstrating an SUV Max of 11.4 indicative of a high-grade malignancy in the left hilum. PET-CT did not demonstrate any other abnormalities to suggest regional nodal disease or wide-spread dissemination. Her case was discussed at a thoracic MDT meeting and a review of images deemed the lesion unsuitable and for biopsy. Due to the highly suspicious nature of the lesion on PET and the patient’s past history of sarcoma, the decision amongst the team was to proceed to a left pneumonectomy.

Routine preoperative work up with an anaesthetist revealed normal lung function tests, cardiac function and blood results of kidney and liver function.

The patient was brought into the operating theatre and a general anaesthetic was administered. Fibre-optic bronchoscopy was performed demonstrating no compression or invasion. A routine left posterolateral thoracotomy was performed. Inspection of the left thoracic cavity revealed no pleural disease. The hilar tumour was palpable and deemed suitable for resection with reasonable margins. The mediastinal pleura was incised and the pericardium opened, with care to avoid injury to the left phrenic nerve. The inferior pulmonary ligament was then divided in order to release the lung, with only the hilum to divide next. Care was taken with minimal manipulation, so as not to embolize the tumour in the left pulmonary vasculature or across to the right side. The left main pulmonary artery was carefully dissected and then divided proximal to the tumour using vascular staplers. Attention was then directed at the pulmonary veins which were divided using vascular staplers. The left main bronchus was then identified and divided with staplers. Once it was clear that the tumour was well within the margins of the specimen, stations 4 L, 5, 6, 7, 8, 9 and 10 lymph nodes were dissected with care to ensure complete lymph node resection. Traction sutures were placed on the pericardium to prevent it from retracting towards the opposite hemithorax. A 14 French pigtail drain was placed in the left pleural cavity. Routine closure and dressings were administered, and the patient extubated on the operating table and transferred to the intensive care unit.

Patient was cleared for the ward and the pleural drain was removed on Day 1, with no significant post-operative issues. She was discharged home on Day 7 with no post-operative complications.

**Figure 1:**
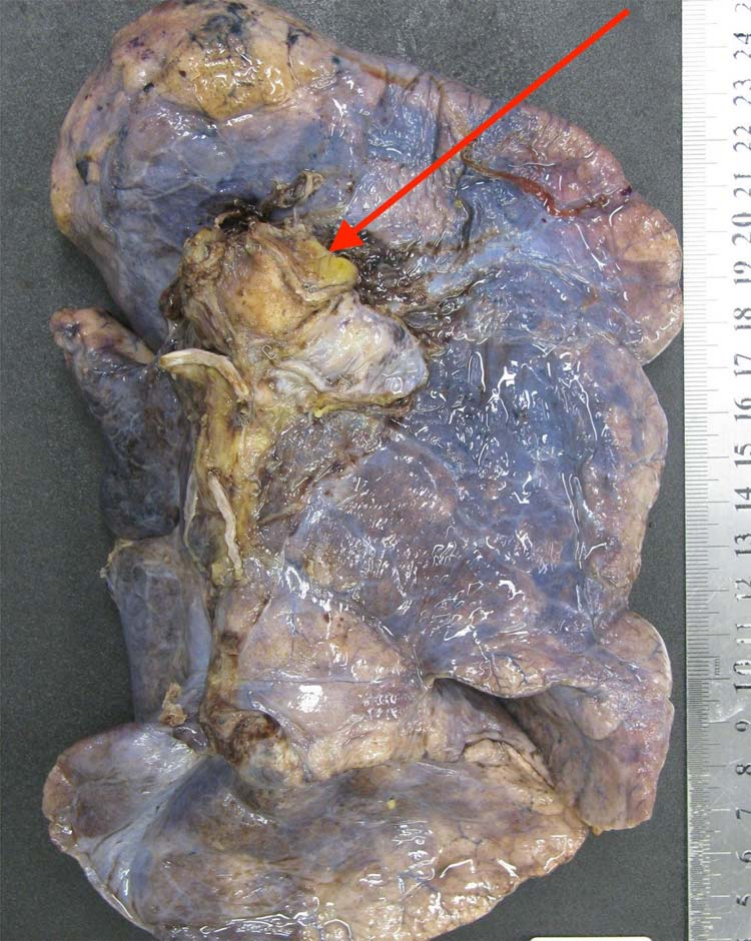
The left lung specimen resected en-bloc with the lesion clearly within the staple line and margins of the pulmonary artery.

**Figure 2:**
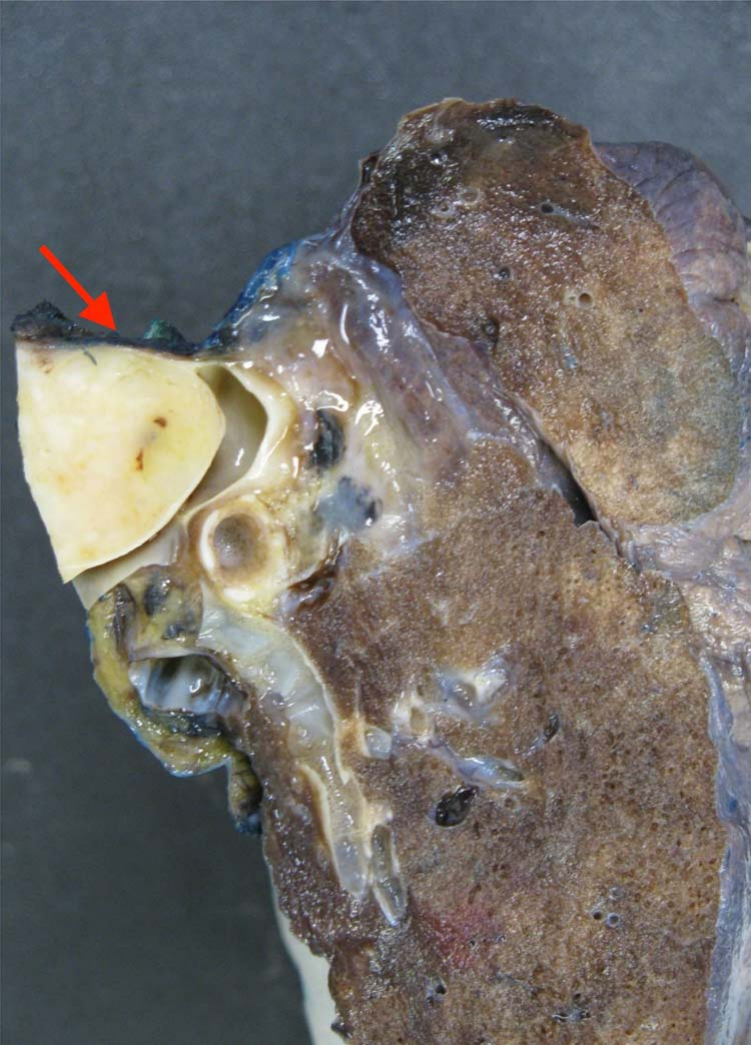
A large cross-section of the specimen demonstrating the significant intravascular obstruction caused by the angiofibromatoid histiosarcoma.

**Figure 3:**
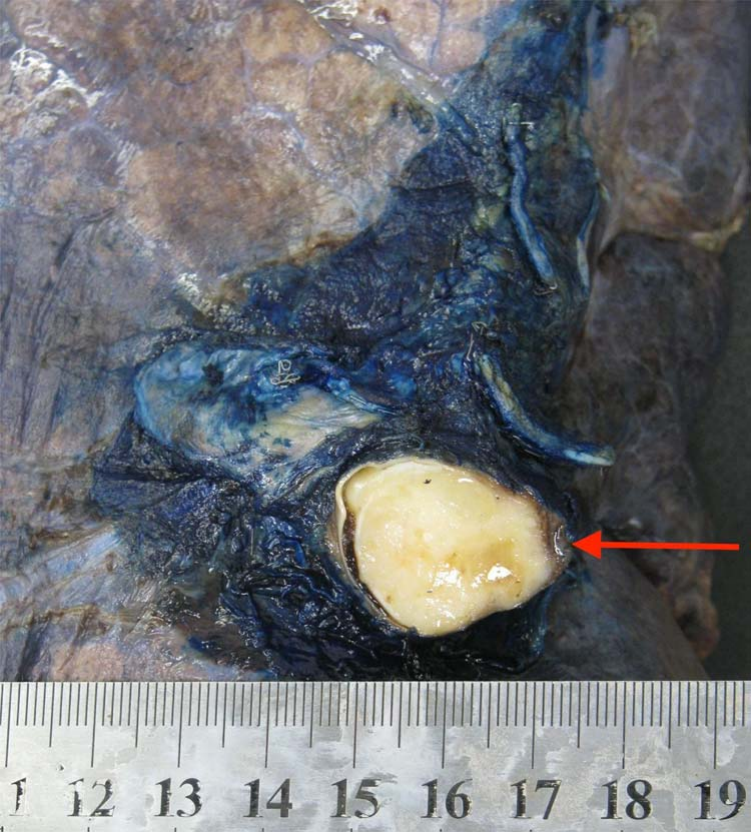
A cross-section image demonstrating the angiofibromatoid histiosarcoma obstructing the left main pulmonary artery.

The histopathology was reviewed by four senior pathologists whom concluded that the results favoured the diagnosis of intravascular angiomatoid fibrous histiocytoma. The tumour itself was 25 × 23 × 20mm in diameter (Figs. 1–3), predominantly being intravascular in nature, although there was a small extravascular component. It extended from close to the staple line margin along the vessel up to 28 mm. Of the lymph node clearance (stations 4 L, 5, 6, 7, 8, 9 and 10), all nodes were free of invasion. The tumour was sent for histological staining and FISH test, and it was determined once again to favour angiomatoid fibrous histiocytoma (EWSR1 arrangement), with no evidence of intimal sarcoma. This is an incredibly rare tumour, in particular in this position (left main pulmonary artery). Her progress and further treatment was discussed in the following MDT meeting, and no further therapy was indicated, with routine surgical follow-up and surveillance.

## DISCUSSION

This case demonstrates a surgical quandary for the management of a large pulmonary vascular lesion. It both demonstrates that these lesions are rarely safe to biopsy preoperatively to determine origin, grade and prognosis. Intravascular lesions have most frequently been diagnosed incidentally following pulmonary vascular endarterectomy [9]. With such few cases described, the natural progression, metastatic nature and overall prognosis with or without surgical intervention is poorly understood. Pneumonectomy carries its own risk profile. At the least, the symptoms of this patient were improved with removal of a near obstructive pulmonary vascular lesion which was acting as a sub-massive pulmonary embolus. Although AFH in the pulmonary artery position has previously been reported once in 2014 [6], the surgical removal was not described in any detail and it is unclear whether or not a pneumonectomy was performed to safely remove the lesion, although it is most likely.

## CONCLUSION

Our case raises the questions on how to both attain preoperative diagnosis, and resect a lesion in this position. We have demonstrated that it is safe and successful to do so with curative intent in the form of a pneumonectomy.
